# Increasing thyroid cancer incidence in Lithuania in 1978–2003

**DOI:** 10.1186/1471-2407-6-284

**Published:** 2006-12-11

**Authors:** Giedre Smailyte, Edita Miseikyte-Kaubriene, Juozas Kurtinaitis

**Affiliations:** 1 Institute of Oncology, Vilnius University, Division of Cancer Prevention, Polocko 2, Vilnius, Lithuania; 2 Institute of Oncology, Vilnius University, Department of Interventional Sonoscopy and Ultrasound Diagnostic, Santariskiu 1, Vilnius, Lithuania; 3 Institute of Oncology, Vilnius University, Cancer Registry, Polocko 2, Vilnius, Lithuania

## Abstract

**Background:**

The aim of this paper is to analyze changes in thyroid cancer incidence trends in Lithuania during the period 1978–2003 using joinpoint regression models, with special attention to the period 1993–2003.

**Methods:**

The study was based on all cases of thyroid cancer reported to the Lithuanian Cancer Registry between 1978 and 2003. Age group-specific rates and standardized rates were calculated for each gender, using the direct method (world standard population). The joinpoint regression model was used to provide estimated annual percentage change and to detect points in time where significant changes in the trends occur.

**Results:**

During the study period the age-standardized incidence rates increased in males from 0.7 to 2.5 cases per 100 000 and in females from 1.5 to 11.4 per 100 000. Annual percentage changes during this period in the age-standardized rates were 4.6% and 7.1% for males and females, respectively. Joinpoint analysis showed two time periods with joinpoint in the year 2000. A change in the trend occurred in which a significant increase changed to a dramatic increase in thyroid cancer incidence rates. Papillary carcinoma and stage I thyroid cancer increases over this period were mainly responsible for the pattern of changes in trend in recent years.

**Conclusion:**

A moderate increase in thyroid cancer incidence has been observed in Lithuania between the years 1978 and 2000. An accelerated increase in thyroid cancer incidence rates took place in the period 2000–2003. It seems that the increase in thyroid cancer incidence can be attributed mainly to the changes in the management of non palpable thyroid nodules with growing applications of ultrasound-guided fine needle aspiration biopsy in clinical practice.

## Background

Thyroid cancers constitute 1% of all malignancies worldwide and are heterogenous in terms of histology, clinical presentation, treatment response and prognosis. The annual incidence of thyroid cancer varies considerably in different registries. High incidence rates in Hawaii, Iceland and Israel have been known for some years. For the most recent data, female Hawaiians, Icelanders, Israeli Jews and Austrian Tyrolese have rates in the range of 8.5 – 19.4 per 100 000 [1]. In Europe, the highest incidence occurs in Iceland, followed by Austria and Finland, while relatively low incidence characterizes the UK, Netherlands and Denmark.

The only established risk factor for thyroid cancer in humans, besides age and gender, is ionizing radiation [2]. Sex hormones, iodine deficiency and other factors have been proposed as risk factors for thyroid cancer, but the findings are inconsistent [3-7].

The incidence of thyroid cancer is increasing in some European countries, USA and Canada [8-11]. The increase in thyroid cancer incidence in Lithuania, observed over the last 20 years, has raised public concern about its association with the Chernobyl nuclear power plant accident in 1986 [12]. Post-Chernobyl studies of thyroid cancer incidence in Belorussia and Ukraine have showed an increased risk, especially those covering population of the most contaminated areas [13]. Thyroid cancer trends in other countries neighboring Belorussia and Ukraine can help validate the risk assessment and explain the rather high country rate variation.

The aim of this study is to analyze changes in thyroid cancer incidence trends in Lithuania during the period 1978–2003 using joinpoint regression models [14].

## Methods

Data on thyroid cancer incidence for the years 1978 through 2003 were obtained from the Cancer Registry of Lithuania (Registry). The Registry covers the entire population of the Republic of Lithuania (3.48 million at the 2001 census) and is located at the national cancer center recently operating as Vilnius University Institute of Oncology, where more that 50% of cancer patients in Lithuania are admitted for treatment. The main sources of data are notifications gathered from all hospitals and diagnostic centers in Lithuania. This information is completed by death certificates stating cancer diagnosis, and by notifications from regional health centers. In 2003, 1.5% of all new cancer cases were registered from death certificates only and 85.7% of diagnoses were confirmed microscopically.

The study was based on all cases of thyroid cancer reported to the Registry during 1978–2003. Corresponding population data, by age, sex and year were available from Department of Statistic of the Republic of Lithuania.

Analyses were based on new cases of invasive thyroid cancer (*International Classification of Disease – Oncology*, 1st edition, site codes 1930–1939 for the period 1978–1997, and *International Classification of Disease – Oncology*, 2nd edition, site codes C730-C739 for the period 1998–2003).

A more detailed analysis by histopathology and stage of disease was performed for the period 1993–2003. Analysis was limited to this period because detailed and reliable information on histology and stage is not available for patients diagnosed before 1993. During the period 1993–2003 the TNM system was used for coding the stage of disease and *International Classification of Diseases – Oncology*, for coding of morphology (1st edition, for the period 1993–1997 and 2nd edition for later period).

Age-specific and age-standardized incidence rates were calculated. Standardization was performed using the direct method (world standard population). Age-standardized rates were calculated for each calendar year, for all ages combined and age-specific rates for age groups 0–19, 20–44, 45–64 and 65 and over.

The joinpoint regression analysis was used to identify points where a statistically significant change over time in linear slope of the trend occurred [14]. The analysis starts with the minimum number of joinpoints, and tests whether one or more joinpoints are statistically significant and should be added to the model. The tests of significance use a Monte Carlo permutation method. In the final model, each joinpoint indicates a statistically significant change in trend, and an estimated annual percentage change (EAPC) is computed for each of those trends by means of generalized linear models assuming a Poisson distribution. A maximum number of 3 joinpoints was allowed for estimations. Joinpoint analysis was performed for all ages combined and age-specific rates for the following age groups 0–19, 20–44, 45–64, and 65 and over. A Joinpoint software version 2.6 was used [15].

## Results

### Changes in thyroid cancer incidence

**Figure 1:**
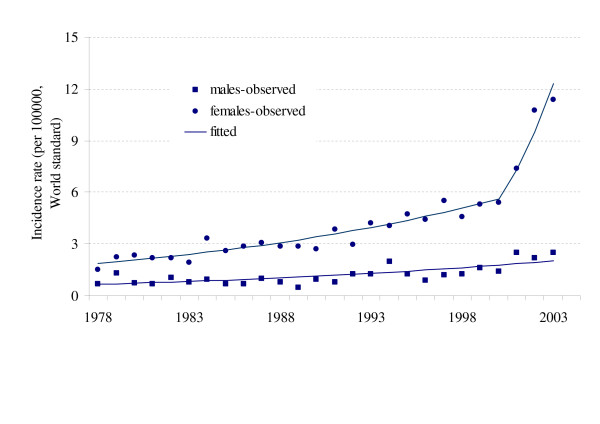
Age-standardised (per 100 000 world standard) thyroid cancer incidence rates in Lithuania, 1998–2003.

In the year 2003 there were 47 thyroid cancer cases in males and 280 in females in Lithuania. This represents 0.6% and 3.7%, respectively, of all new malignant tumours. Incidence rates were considerably lower for males than for females, with male:female ratio 1:3. Thyroid cancer incidence rates in males changed during the study period from 0.7/100 000 in 1978 to 2.5/100 000 in the year 2003, and from 1.5/100 000 to 11.4/100 000 in females (Figure 1).

**Figure 2:**
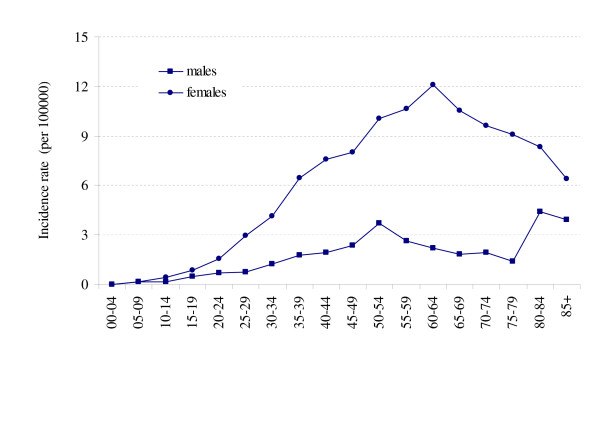
Age-specific thyroid cancer incidence rates in Lithuania, 1978–2003.

Age-specific thyroid cancer incidence rates are shown in Figure 2. In females thyroid cancer incidence increases steadily up to 65 years of age, and decreases thereafter. Little change in the male's thyroid cancer age-specific incidence rates was observed.

Results of the joinpoint analysis of incidence time trends are shown in Table 1. Annual percentage changes in the age-standardized rates over this period were 4.6% and 7.1% for males and females, respectively, for all thyroid carcinomas combined.

Joinpoint analysis does not show the existence of statistically significant points of change in male thyroid cancer incidence. Age group analysis revealed that for the entire study period, rates increased significantly in almost all age groups except one. In age group 20–44 incidence started to increase by 29.7% per year since 1999.

In females, after a moderate increase of 5.2% per year until 2000, a sharp increase in incidence occurred, reaching 29.8% per year in recent years. The year 2000 was recognized as a statistically significant change point in incidence trend for the age group 20–44 years. In the age group 45–64 years, moderate increase was followed by a dramatic increase in period 1999–2003.

Thyroid cancer incidence analysis by sex and age group revealed that for the entire study period rates increased significantly in both sexes and in almost all age groups. However, joinpoint analysis makes it possible to distinguish two time periods: an initial period of statistically significant increase and a second period of a dramatic rate increase. In the year 2000, a change in females thyroid cancer incidence trend occurred in the age group 20–44 years and overall rates in which a significant increase changed to an accelerated increase. The same pattern of increase was observed for males in the age group 20–44 and for females in the age group 45–64 years with joinpoint in year 1999.

### Changes in thyroid cancer incidence by histopathology and by stage in 1993–2003

Results of the joinpoint analysis of trends by histopathology and stage of disease, for both sexes combined, are shown in Table 2. Also there were changes in thyroid cancer incidence rates, with increases in papillary and follicular carcinoma. Estimated annual percentage changes over this period were 11.4% for all thyroid carcinomas combined; 17.7% for papillary carcinoma and 9.2% for follicular carcinoma. A statistically significant change in trend was found in papillary and follicular thyroid carcinoma incidence in 1999 and 2001 years respectively.

Joinpoint analysis by stage of disease showed the highest increase in I stage thyroid cancer, with statistically significant change in trend in the year 2000.

## Discussion

In Lithuania, as in many other countries, the incidence of thyroid cancer is increasing. Most probably, the trends reflect an environmental risk increase that is boosted by augmented diagnostic activity following more careful pathological examination.

The increase in thyroid cancer risk could be attributed to ionizing radiation exposure [2]. Environmental and occupational exposures from the Chernobyl accident were studied in Lithuania – the recent estimates do not confirm an evident contribution to thyroid cancer rates for the Lithuanian population. In a study of Lithuania clean-up workers, no elevated thyroid cancers risk due to exposure was detected. There was no association with the level of radiation dose or duration of stay in the area of Chernobyl [16]. Studies of individuals living in the Chernobyl areas have shown an increased risk among those exposed as children [13]. In Lithuania in 1987 thyroid cancer incidence rates for males and females, in age group 0–19 years were 0.2 and 0.6 per 100 000 and in 2003 0.9 and 0.6 respectively. Ionizing radiation exposure, therefore cannot explain the recent increase in incidence of thyroid cancer in Lithuania; other factors should have contributed importantly to the changing pattern.

In addition to this well-known risk factor of thyroid cancer, the increase of diagnostic activity has been suspected to be of etiological importance in the observed increase of thyroid cancer incidence. Analysis of the impact of changes in medical practice found an association between the spread of fine-needle aspiration biopsy and the increase in thyroid cancer incidence [17]. In Lithuania, the overall thyroid cancer increase was caused primarily by an increase in papillary thyroid cancer and in I stage thyroid cancer. Ultrasound guided fine-needle aspiration biopsy of thyroid nodules was first introduced in Lithuania in 1997. At about at the same time, the practice of follow-up of small thyroid nodules was changed accordingly. The method of ultrasound guided aspiration biopsy of asymptomatic thyroid nodules smaller than 1 cm in diameter was employed for the follow-up. If one takes into consideration the dynamic of fine-needle aspiration biopsy dissemination in the region, then the increase in thyroid cancer incidence would seem to be correlated well with the new diagnostic technology.

Studies from other countries confirm our suggestions of the possible impact of changes in the management of thyroid nodules and diagnostic activities. Increase in papillary thyroid cancer incidence in Geneva has been found to be related mainly to changes in histological diagnostic criteria, and, to a lesser extent, to increased diagnostic activity [18]. The proportion of microcarcinomas and silent carcinomas increased from 17% to 24% between 1970–79 and 1990–98. A multicentric study in France showed a significant increase, from 1980 to 2000, in ultrasonographic (from 3 to 84.8%) and fine-needle aspiration biopsy (from 4,5 to 23%) of patients with thyroid nodules as well as a significant association between the increase in the prevalence of thyroid carcinomas among operated patients (from 12.5 to 37) [17]. There was increased incidence of thyroid cancer in France, mainly due to papillary type, with an epidemic of microcarcinomas (43% of operated cancers, for the period 1998–2001) [9].

The increasing incidence of thyroid cancer in the United States also was related to the increased detection of small papillary cancers. Between 1988 and 2002, 49% of the increase consisted of cancers measuring 1 cm or smaller; 87% consisted of cancers measuring 2 cm or smaller [10]. The authors suggested that increasing incidence with stable mortality rates reflects increased detection of subclinical disease, not an increase in the true occurrence of thyroid cancer. Most probably, increased thyroid cancer incidence rates in Lithuania may also be attributed to diagnostic improvements and, consequently, to the discovery of smaller tumours.

The joinpoint analysis has recognized evident changes only by years 1999 and 2000 that can be a reflection of major changes in diagnostic and treatment paradigms and new diagnostic technology in the country. The results show changes in thyroid cancer incidence, and the relative importance of the diagnostic activities in recent years. Additional research on the risk factors for thyroid cancer and incidence changes related to diagnostic practices is needed to explain the peaking of the incidence rates in Lithuania.

## Conclusion

A moderate increase in thyroid cancer incidence has been observed in Lithuania between the years 1978 and 2000. An accelerated increase in thyroid cancer incidence rates took place in the period 2000–2003. It seems that the increase in thyroid cancer incidence can be attributed mainly to the changes in the management of non palpable thyroid nodules with the growing application of ultrasound-guided fine needle aspiration biopsy in clinical practice.

## Competing interests

The author(s) declare that they have no competing interests.

## Authors' contributions

GS was responsible for the development of intellectual content and the study design, statistical analyses, interpretation of the results and manuscript drafting. EMK was responsible for interpretation of the results and manuscript drafting. JK was responsible for the development of intellectual content and the critical revisions of manuscript. All authors read and approved the final manuscript.

## Pre-publication history

The pre-publication history for this paper can be accessed here:



## Figures and Tables

**Table 1:** Thyroid cancer incidence rates and joinpoint analysis (1978–2003).

	1978		2003		EAPC 1978–2003	Trend 1		Trend 2	
			
	N	Rate	N	Rate		Years	EAPC	Years	EAPC
Males									
Age (years)									
00–19	0	0.0	4	0.9	4.8*	1978–2003	4.8*		
20–44	4	0.7	26	4.2	5.8*	1978–1999	2.5	1999–2003	29.7*
45–64	5	1.7	12	3.4	4.9*	1978–2003	4.9*		
65+	1	0.7	5	2.9	2.2	1978–2003	2.2		
Standardized	10	0.7	47	2.5	4.6*	1978–2003	4.6*		
(95% PI)		(0.2–1.1)		(1.8–3.2)					

Females									
Age (years)									
00–19	0	0.0	2	0.4	1.2	1978–2003			
20–44	8	1.3	82	12.9	7.3*	1978–2000	5.1*	2000–2003	31.5*
45–64	13	3.3	141	32.7	9.3*	1978–1999	6.5*	1999–2003	26.9*
65+	15	6.3	55	16.4	3.7*	1978–2003			
Standardized	36	1.5	280	11.4	7.1*	1978–2000	5.2*	2000–2003	29.8*
(95% PI)		(1.0–2.0)		(10.0–12.8)					

*The EAPC is significantly different from 0 (*P *< 0.05).

EAPC estimated annual percentage change.

**Table 2:** Thyroid cancer joinpoint analysis by stage and histopathology (1993–2003).

	1993		2003		EAPC 1978–2003	Trend 1		Trend 2	
			
	N	Rate	N	Rate		Years	EAPC	Years	EAPC
All cases	128	3.5	325	9.4	11.4*	1993–2000	5.3*	2000–2003	27.5*
									
Histopathology									
papillary	58	1.6	249	7.2	17.7*	1993–1999	6.2	1999–2003	33.2*
follicular	16	0.4	47	1.4	9.2*	1993–2001	4.7	2001–2003	32.3
other	54	1.5	29	0.8	-2.4	1993–2003	-2.4		

Stage									
I	12	0.3	162	4.7	29.6*	1993–2000	14.6	2000–2003	58.9*
II	66	1.8	66	1.9	3.5	1993–2003	3.5		
III	19	0.5	80	2.3	17.1*	1993–2003	17.1*		
IV	20	0.5	16	0.5	5.0	1993–2001	10.5*	2001–2003	-33.1
unspecified	11	0.3	1	0.03	-18.6*	1993–2003	-18.6*		

*The EAPC is significantly different from 0 (*P *< 0.05).

EAPC – estimated annual percentage change.
